# Response to Comment by Dr. Kuang on Our Manuscript “Immediate Oral Refeeding in Patients With Mild and Moderate Acute Pancreatitis: A Multicenter, Randomized Controlled Trial (PADI trial)”

**DOI:** 10.1097/AS9.0000000000000472

**Published:** 2024-07-18

**Authors:** Elena Ramírez-Maldonado, Sandra López Gordo, Robert Memba, Rosa Jorba

**Affiliations:** * From the General and Digestive Surgery Department, Joan XXIII University Hospital of Tarragona, Rovira i Virgili University, Tarragona, Spain; † General and Digestive Surgery Department, Maresme Health Consortium, Mataro, Spain.; Hepato-Pancreato-Biliary and Transplant Surgery Department, Vall d’Hebron University Hospital, Barcelona, Spain; Maresme Health Consortium, Matarò, Spain; Vall d’Hebron University Hospital, Barcelona, Spain; University Hospital of Tarragona Joan XXIII, Rovira i Virgili University, Tarragona, Spain; Althaia University Hospital, Xarxa Asistencial Universitària de Manresa, Sant Joan de Déu Hospital, Manresa, Spain; Moise’s Broggi Hospital, CSI, Barcelona, Spain; Barcelona Clinic Hospital, IDIBAPS, CIBEREHD, University of Barcelona, Barcelona, Spain; University Hospital of Tarragona Joan XXIII, Rovira i Virgili University, Tarragona, Spain; Maresme Health Consortium, Matarò, Spain; University Hospital of Tarragona Joan XXIII, Rovira i Virgili University, Tarragona, Spain; University Hospital of Tarragona Joan XXIII, Rovira i Virgili University, Tarragona, Spain; University Hospital of Tarragona Joan XXIII, Rovira i Virgili University, Tarragona, Spain; Moise’s Broggi Hospital, CSI, Barcelona, Spain; University Hospital of Tarragona Joan XXIII, Tarragona, Spain; Clinical Nutrition Department, The Third People’s Hospital of Chengdu, Affiliated Hospital of Southwest Jiaotong University, Chengdu, China; University Hospital of Tarragona Joan XXIII, Rovira i Virgili University, Tarragona, Spain; Hospital Garcia de Orca EPE, Almada, Portugal; Robert Memba, University Hospital of Tarragona Joan XXIII, Rovira i Virgili University, Tarragona, Spain; Barcelona Clinic Hospital, IDIBAPS, CIBEREHD, University of Barcelona, Barcelona, Spain; Vall d’Hebron University Hospital, Barcelona, Spain; University Hospital of Tarragona Joan XXIII, Rovira i Virgili University, Tarragona, Spain; The Third People’s Hospital of Chengdu, Affiliated Hospital of Southwest Jiaotong University, Chengdu, China; University Hospital of Tarragona Joan XXIII, Tarragona, Spain; Barcelona Clinic Hospital, IDIBAPS, CIBEREHD, University of Barcelona, Barcelona, Spain; Vall d’Hebron University Hospital, Barcelona, Spain; Barcelona Clinic Hospital, IDIBAPS, CIBEREHD, University of Barcelona, Barcelona, Spain; Barcelona Clinic Hospital, IDIBAPS, CIBEREHD, University of Barcelona, Barcelona, Spain; Gastroenterology Department, The Third People’s Hospital of Chengdu, Affiliated Hospital of Southwest Jiaotong University, Chengdu, China; The Third People’s Hospital of Chengdu, Affiliated Hospital of Southwest Jiaotong University, Chengdu, China; The Third People’s Hospital of Chengdu, Affiliated Hospital of Southwest Jiaotong University, Chengdu, China

Acute pancreatitis (AP) guidelines recommend that endoscopic retrograde cholangiopancreatography (ERCP) is not indicated in predicted mild biliary AP without cholangitis (grade 1A, strong agreement) or in severe AP without cholangitis (grade 1B, strong agreement).^1^ Acute cholangitis is a clear indication for urgent ERCP (<24 hours) in patients with AP.^1–4^

Fortunately, many gallstones pass into the duodenum, and only a minority of patients present with choledocholithiasis, which causes biliary obstruction and possible complications derived from this obstruction. The guidelines clearly establish that the main indication for ERCP in patients with AP is sepsis due to acute cholangitis and that ERCP must be performed in the first 24 hours, which contributes to a decrease in morbidity and mortality.^1–3^

In patients with mild AP of biliary origin, with suspicion of choledocholithiasis but with good recovery, no benefit has been seen from performing ERCP before cholecystectomy. To diagnose choledocholithiasis in the presence of suggestive symptoms, imaging tests such as endoscopy ultrasound or magnetic resonance cholangiopancreatography can be performed.^1–5^ Guidelines recommend against the routine use of ERCP in the case of acute cholangitis.^1–4^

In our study on diet in mild and moderate acute pancreatitis (PADI trial),^6^ no cases of AP and cholangitis were reported. The diagnosis of bile duct obstruction was made using magnetic resonance cholangiopancreatography in patients with high clinical suspicion of choledocholithiasis. There were 6 patients in total: 5 underwent ERCP at 2 to 4 weeks after diagnosis and 1 patient underwent a cholecystectomy and bile duct approach within a month of diagnosis.

Patients in the immediate oral refeeding (IORF) group were informed that they would receive a low-fat diet and were encouraged to eat as much as they could. If they experienced pain, nausea, or vomiting, treatment was prescribed, and food was provided. If their intake was ≤50% of the dishes, it was classified as diet intolerance. This part study was observational.

Nonetheless, diet tolerance among patients with IORF was 99%. Initially, a diary was provided to each patient at the promoting center to record any signs or symptoms. However, the responses and tolerance were so positive that this practice was discontinued and maintained only in patients who did not tolerate the diet.

ERCP had no effect on the length of hospital stay. Patients were treated after being discharged from the study, as explained in the methods section: “In cases of hospital length-of-stay prolongation due to cholecystectomy scheduling, medical conditions independent of AP itself, or those waiting for a convalescence center space, the discharge date was instead established by meeting the medical criteria for discharge.”

The management of each patient followed the recommendations of evidence-based guidelines of the International Association of Pancreatology and the American Pancreatic Association.^1^ Discrepancies in adherence to guideline recommendations for the diagnosis and management of AP included both the underutilization and overutilization of resources and treatments.^7^

Therefore, treatment and diet administration were not differentiated by etiology. Upon admission, etiology was determined using personal history, laboratory serum tests, and right upper quadrant ultrasonography. If the AP was idiopathic, a further workup was conducted to investigate its etiology.

It is difficult to determine whether early refeeding prevented the occurrence of progression or whether this result was coincidental. However, a recent evidence-based review of nutritional support in AP found that fasting can lead to intestinal atrophy, loss of epithelial barrier function, and changes in intestinal flora. These changes may trigger a systemic inflammatory response in some patients, increasing the risk of sepsis and organ failure.^8,9^ Current clinical guidelines^1–4^ recommend that oral refeeding should begin early under certain conditions, such as the absence of pain and improvement in laboratory parameters. The PADI trial showed that the IORF group experienced fewer complications than the conventional oral refeeding group, indicating that IORF is safe and feasible in patients with mild and moderate AP.

In addition to the benefits of early refeeding in patients with acute pathologies such as AP, a recent meta-analysis showed that early oral refeeding may be beneficial for patients with mild to moderate AP. This meta-analysis included the PADI trial.^10^

The recommendations of evidence-based guidelines on nutritional support for AP patients include recommendations based on the disease severity. In mild AP patients, although the conditions defining the ideal time to start refeeding are highly variable, the “early” oral diet is preferred, and the PADI demonstrates that IORF is safe and feasible.

## ACKNOWLEDGMENTS

We thank Dr. Yu, Dr. Shen, Dr. Xie, and Dr. Kuang for their pertinent editorial comment on our paper.

E.R.-M. and all authors participated in all phases of the letter. S.L.G., R.M., and R.J.: critical revision of the letter for important intellectual content. All authors read and approved the final letter.

The Catalan Pancreatitis Collaborative Group: Sonia Babiloni, University Hospital of Tarragona Joan XXIII, Tarragona, Spain; Laia Blanco, Hepato-Pancreato-Biliary and Transplant Surgery Department, Vall d’Hebron University Hospital, Barcelona, Spain; Sandra M. Bacca, Maresme Health Consortium, Matarò, Spain; Joaquim Balsells, Vall d’Hebron University Hospital, Barcelona, Spain; Carme Boqué, University Hospital of Tarragona Joan XXIII, Rovira i Virgili University, Tarragona, Spain; Pablo Collera Ormazabal, Althaia University Hospital, Xarxa Asistencial Universitària de Manresa, Sant Joan de Déu Hospital, Manresa, Spain; Daniel Coronado Llanos, Moise’s Broggi Hospital, CSI, Barcelona, Spain; Ignasi Elizalde, Barcelona Clinic Hospital, IDIBAPS, CIBEREHD, University of Barcelona, Barcelona, Spain; Laia Estalella, University Hospital of Tarragona Joan XXIII, Rovira i Virgili University, Tarragona, Spain; Maria Teresa Fernández Planas, Maresme Health Consortium, Matarò, Spain; Lidia Florit Serra, University Hospital of Tarragona Joan XXIII, Rovira i Virgili University, Tarragona, Spain; Inmaculada Fonoll, University Hospital of Tarragona Joan XXIII, Rovira i Virgili University, Tarragona, Spain; Nil Gómez Vallvé, University Hospital of Tarragona Joan XXIII, Rovira i Virgili University, Tarragona, Spain; Sergio González, Moise’s Broggi Hospital, CSI, Barcelona, Spain; Maria Alejandra Guerrero Ortíz, University Hospital of Tarragona Joan XXIII, Tarragona, Spain; Jiazhen Li, Clinical Nutrition Department, The Third People’s Hospital of Chengdu, Affiliated Hospital of Southwest Jiaotong University, Chengdu, China; Erik Llàcer-Millán, University Hospital of Tarragona Joan XXIII, Rovira i Virgili University, Tarragona, Spain; Rui Pedro Major Branco, Hospital Garcia de Orca EPE, Almada, Portugal; Robert Memba, University Hospital of Tarragona Joan XXIII, Rovira i Virgili University, Tarragona, Spain; David Nicolas, Barcelona Clinic Hospital, IDIBAPS, CIBEREHD, University of Barcelona, Barcelona, Spain; Elizabeth Pando, Vall d’Hebron University Hospital, Barcelona, Spain; Mihai-Calin Pavel, University Hospital of Tarragona Joan XXIII, Rovira i Virgili University, Tarragona, Spain; Jing Quiao, The Third People’s Hospital of Chengdu, Affiliated Hospital of Southwest Jiaotong University, Chengdu, China; Marta Rodrigo-Rodrigo, University Hospital of Tarragona Joan XXIII, Tarragona, Spain; Ariadna Sánchez, Barcelona Clinic Hospital, IDIBAPS, CIBEREHD, University of Barcelona, Barcelona, Spain; Teresa Soriano, Vall d’Hebron University Hospital, Barcelona, Spain; Guillem Soy, Barcelona Clinic Hospital, IDIBAPS, CIBEREHD, University of Barcelona, Barcelona, Spain; Eva Vaquero, Barcelona Clinic Hospital, IDIBAPS, CIBEREHD, University of Barcelona, Barcelona, Spain; Pengyu Wu, Gastroenterology Department, The Third People’s Hospital of Chengdu, Affiliated Hospital of Southwest Jiaotong University, Chengdu, China; Heng Xi, The Third People’s Hospital of Chengdu, Affiliated Hospital of Southwest Jiaotong University, Chengdu, China; Wen Zhong, The Third People’s Hospital of Chengdu, Affiliated Hospital of Southwest Jiaotong University, Chengdu, China.
